# Improving *E. coli* growth performance by manipulating small RNA expression

**DOI:** 10.1186/s12934-017-0810-x

**Published:** 2017-11-14

**Authors:** Alejandro Negrete, Joseph Shiloach

**Affiliations:** 1 Biotechnology Core Laboratory, NIDDK, NIH, Bethesda, MD 20892 USA; 2 Present Address: MilliporeSigma, Carlsbad, CA 92009 USA

**Keywords:** Small RNA, Stress response, High cell density cultures, *E. coli*, Acetate production

## Abstract

Efficient growth of *E. coli*, especially for production of recombinant proteins, has been a challenge for the biotechnological industry since the early 1970s. By employing multiple approaches, such as different media composition, various growth strategies and specific genetic manipulations, it is now possible to grow bacteria to concentrations exceeding 100 g/L and to achieve high concentrations of recombinant proteins. Although the growth conditions are carefully monitored and maintained, it is likely that during the growth process cells are exposed to periodic stress conditions, created by fluctuations in pH, dissolved oxygen, temperature, glucose, and salt concentration. These stress circumstances which can occur especially in large volume bioreactors, may affect the growth and production process. In the last several years, it has been recognized that small non-coding RNAs can act as regulators of bacterial gene expression. These molecules are found to be specifically involved in *E. coli* response to different environmental stress conditions; but so far, have not been used for improving production strains. The review provides summary of small RNAs identified on petri dish or in shake flask culture that can potentially affect growth characteristics of *E. coli* grown in bioreactor. Among them MicC and MicF that are involved in response to temperature changes, RyhB that responds to iron concentration, Gady which is associated with lower pH, Sgrs that is coupled with glucose transport and OxyS that responds to oxygen concentration. The manipulation of some of these small RNAs for improving growth of *E. coli* in Bioreactor is described in the last part of the review. Overexpression of SgrS was associated with improved growth and reduced acetate expression, over expression of GadY improved cell growth at acidic conditions and over expression of OxyS reduced the effect of oxidative stress. One of the possible advantages of manipulating sRNAs for improving cell growth is that the modifications occur at a post-translational level. Therefore, the use of sRNAs may exert minimal effect on the overall bacterial metabolism. The elucidation of the physiological role of newly discovered sRNAs will open new possibilities for creating strains with improved growth and production capabilities.

## Background

*Escherichia coli* is the preferred platform for industrial production of various biological products due to its short doubling time, ability to grow to high cell densities, the relatively simple scale-up procedure, cost-effectiveness, known genomics, as well capability to express high concentrations of recombinant proteins [1–4]. Of the 94 protein-based anti-cancer pharmaceuticals currently on the market, 70% are produced in *E. coli* [5, 6].

Production of biologicals from *E. coli* is typically done by growing the microorganism to high density, a procedure that was developed in the mid 1970s [7], and is currently the method of choice [6, 8]. Achieving high cell concentration of the bacteria is done by adequate supply of nutrients, such as carbon sources (mostly glucose) and dissolved oxygen and by controlling growth parameters such as pH and temperature. Although the growth parameters are being controlled, fluctuations in osmolality, temperature, dissolved oxygen, substrates, and metabolites concentration such as glucose, acetate and ammonia can affect bacterial growth and production capability [9]. Bacterial strains resistant to stress are expected to exhibit improved growth and recombinant protein production. Genetic engineering approaches have generated strains resistant to stress [8, 10–13]. However, the genetic modifications were usually associated with lower cell concentrations or production of metabolites that affect the overall process efficiency [8, 11]. *E. coli* responds to different stress parameters by initiating responses through the activation of complex physiological and molecular mechanisms [14, 15]. In the past few years, it has been recognized that small RNAs play a role as regulators of bacterial gene expression [16, 17]. These molecules were found to be involved in *E. coli* response to different environmental stresses [18–20]. About 100 sRNAs were discovered by microarrays, biocomputational prediction algorithms and confirmed by reporter gene tags, northern blots, or pulse-expression [21–23]. The functions of only a few have been identified by experimental methods such as specific binding of the sRNA to its target, verifying the stress conditions that triggered its expression, and showing the evidence of its physiological effect [24]. In this review, we are focusing on those small RNAs that can potentially enhance bacterial response to stress especially when grown to high cell densities in bioreactors.

## Small RNAs respond to stress during *E. coli* growth in shake flasks

Most of the information on sRNAs involvement in response to stressed growth conditions was obtained from *E. coli* growing to low density in shake flask. The data obtained from these studies identified several sRNAs responding to stress including temperature, osmolarity, iron limitation, glucose phosphate accumulation, pH, and oxygen. All these studies concentrated only on understanding the molecular response of the sRNA to the stress, but not on the possible physiological implications of manipulating the expression of these sRNA. The information currently known is summarized in Table 1.

**Table 1:** Hfq-dependent sRNAs involved in the stress response of *E. coli* growing in bioreactor

Stressor	RNA	Length	Regulation	Physiological response	References
Temperature	MicC	109	Increased at low temperature	Repression of porin synthesis OmpC	[25]
	MicF	93	Increased at high temperature	Repression of translation and stability of porin OmpF	[26]
	DsrA	87	Increased at low temperature	Activation of translation of rpoS and inhibition of translation of hns	[27–30]
Osmolarity	MicF	93	Induced by high osmolarity	Repression of translation and stability of porin OmpF	[31, 32]
	RprA	105	Induced by high osmolarity	Activation of translation of rpoS	[32–34]
Iron	RyhB	90	Induced by limited iron	Repression of iron enzymes, TCA cycle and respiratory chain	[35, 36]
Oxygen	OxyS	109	Induced by oxidative stress (H_2_O_2_); activated by OxyR	Repression of unneeded activities and protection against mutagenesis	[37]
	FnrS	113	Induced under anaerobic conditions; activated by FNR, ArcA and CRP	Repression of genes from anaerobic metabolism and oxidative stress	[38, 39]
Glucose	SgrS	227	Induced by glucose-phosphate or analogs; activated by SgrR	Prevent sugar uptake, reduce accumulated sugar-phosphates by repressing *ptsG* synthesis	[40, 41]
	CyaR	87	Induced by low glucose; activated by CRP	Repression of porin synthesis, membrane proteins, and synthetase enzymes	[42]
	Spot42	109	Induced by glucose; repressed by CRP	Decrease galactose utilization and non-preferred carbon source metabolism	[43, 44]
pH	GadY	105, 90, 59	Induced by low pH	Activation of acid resistance genes from the GDS	[45, 46]
	RprA	105	Induced by low pH	Activation of translation of rpoS	[47]
	DsrA	87	Increased at low pH	Activation of translation of rpoS	[47, 48]

### Temperature

Two sRNAs, MicC and MicF, were identified to be involved in the regulation of translation and stability of porins [49]. Both were found to be associated with response to temperature changes. MicC was expressed at low temperature (24 °C) and was identified by growing *E. coli* K-12 (JM109) in LB and in M9-glycerol minimal media at different temperatures at a stationary and at an exponential phases to an OD_600_ of 0.2 or 0.4 [25]. MicF was found to be expressed at higher temperatures (37 or 42 °C) and was identified by growing *E. coli* K-12 JA221 overnight to an OD_550_ of 0.2 which was followed by increasing the growth temperature to 37 °C by adding equal volume of medium at 50 °C [32]. Another sRNA responding to temperature change is DsrA, this sRNA was found to be induced in *E*. *coli* K-12 cultures grown in LB media at 24 °C to an OD_600_ of 0.4–0.6 [27]. DsrA is a sRNA that regulates *rpoS* and *hns* mRNAs [29]. This sRNA interacts with the mRNA of the transcriptional regulator σ^S^ (encoded by *rpoS*) and the transcriptional repressor H-NS [50], it activates RpoS translation and inhibits *hns* translation [29, 30].

### Osmolarity

The sRNA MicF, in addition to its response to temperature change, has also been associated with high osmolarity conditions created by adding 20% sucrose to the growth media. MicF expression was increased when *E. coli* K-12 was grown in LB, low-phosphate medium, or in nutrient broth or in M9 medium supplemented with 0.4% glucose, MicF expression was increased when the culture was exposed to high osmolarity conditions [26].

Another sRNA responding to changes in osmolarity is RprA, a sRNA of 105nt that activates RpoS translation when the bacteria are exposed to osmotic shock [33, 34]. Experiments were performed by growing *E. coli* K-12 (MG1655) in LB to stationary phase (OD_600_ = 3.5) and the addition of 0.12, 0.23, 0.46 or 1.0 M sucrose for 20 min [34]. RprA expression has also been induced by osmotic shock in an *E. coli* K-12 MC1061 strain grown in LB to an OD_600_ of 0.3 by adding 0.46 M sucrose [33].

### Iron concentration

The transcription of RyhB, a 90 nt sRNA, was found to be associated with iron availability in the media. RyhB is usually repressed at iron-rich conditions [51] and is expressed when the iron concentration is low, thereby reducing iron consumption by downregulating the expression of iron-containing proteins and, as a result, increasing the concentration of free intracellular Fe^3+^ [51, 52]. This was studied by using derivatives of *E. coli* K-12 MG1655 that were grown in M63 media supplemented with 1 μM FeSO_4_ [52]. Other studies to access the effect of high iron concentration were conducted by growing *E. coli* K-12 MG1655 to an OD_600_ = 0.3 in LB or M63 media supplemented with 0.2% glycerol at 50 μM FeSO_4_ [35]. These showed that increased RyhB expression triggered reduced expression of genes associated with the TCA cycle and the respiratory chain such as *acnA* (aconitase), *sdhCDAB* (succinate dehydrogenase), *sodB* (superoxide dismutase), *fumA* (fumarase), and ferritins *bfr* and *ftnA* [35, 36].

### pH

The sRNA GadY has been linked with increased expression of genes associated with response to acidic conditions. There are three species of the GadY sRNA: full-length GadY at 105 nt and two processed forms at 90 and 59 nt respectively; all detected when *E. coli* K-12 MC4100 and *E. coli* K-12 MG1655 were grown in shake flasks in LB at pH 5.8 [45]. In a different experiment, GadY and its processed forms were detected in in *E. coli* grown to OD_600_ of 2.0 in LB-MES media buffered at pH 5.5 with 100 mM 2-(N-morpholino)ethanesulfonic acid (MES) [46].

Overexpression of GadY was associated with activation of the acid resistance genes *gadA*, *gadB*, and *gadC* (part of the GDS-glutamate decarboxylase system), which in turn activated the GadX mRNA, inducing the expression of GDS [45, 53, 54]. GadY was also found to activate the Lysine decarboxylase system (LDS) at pH 5.8 [55]. Two other sRNAs RprA and DsrA have been associated with acid resistance [47]. The RprA and DsrA were expressed when *E. coli* K-12 MG1655 cells were grown in M9 media supplemented with 0.4% glucose. The culture was diluted 1:10 or 1:100 with M9 minimal media supplemented with 0.4% glucose and 1.5 mmol/L glutamate, which was adjusted to pH 2.0 or 3.0 with concentrated HCl [47]. RprA induces the expression of *rpoS* which protects against acid resistance [47]. The overexpression of DsrA induces *rpoS* similarly to the RprA sRNA [48].

### Glucose transport and glucose phosphate accumulation

Expression of the glucose transporter ptsG was found to be regulated at the posttranscriptional level by the 227 nt sRNA SgrS that binds to the mRNA *ptsG* [40, 56]. The effect of SgrS was observed when *E. coli* K-12 (MG1655) grew in M63 minimal media agar plates supplemented with 0.2% glucose, and in liquid LB medium supplemented with 1% of glucose or the non-metabolized glucose alpha methyl glucoside (αMG) [40, 57]. It was established that SgrS is expressed in response to glucose-6-phosphate accumulation, inhibiting the translation of the *ptsG* mRNA, and as result causing decrease of the glucose transporter IICB^Glc^ and glucose entry into the cells [58–61]. SgrS also encodes the 43 amino acids polypeptide SgrT that regulates the activity of pre-existing IICB^Glc^ transporters [41]. This polypeptide rescues cells growing in the presence of αMG by reducing glucose transport independently of mRNA degradation [41, 62]. Other sRNAs that are associated with carbon metabolism are CyaR and Spot42. CyaR an 87 nt sRNA, is induced at low glucose concentrations and is positively regulated by the global regulator Crp [42]. This was studied by growing *E. coli* in shake flask or agar plates containing Lennox broth or M63 minimal medium supplemented with 0.001% vitamin B1 and 0.2% glucose or glycerol [42]. Overexpression of CyaR downregulated at least 25 additional genes, responsible for the expression of membrane proteins, transporters, and essential enzymes to adapt cells to low glucose conditions [42]. Spot42 is a109 nt Hfq-dependent sRNA that represses genes involved in central and secondary metabolism, redox balancing, and consumption of various non-preferred carbon sources resulting in slow growth [63]. The information above was obtained by growing *E. coli* K-12 in LB or M9 media supplemented with 10 μg/mL thiamine, 2 mM MgSO_4_, 0.1 mM CaCl_2_, and 0.2% casamino acids, and different carbon sources [63].

### Oxygen

The sRNA OxyS, is induced in response to increased expression of the OxyR regulon which was triggered by exposing *E. coli* K-12, grown in LB media to OD_600_ = 0.2, to H_2_O_2_ [37]. OxyS acts post-transcriptionally by inhibiting rpoS and regulating the expression of FhlA (format hydrogen lyase activator) which protects the cells against mutagenesis [37, 64, 65].

High oxygen concentrations cause cellular damage by affecting enzymes, proteins, and DNA through the formation of reactive oxygen species (ROS). *E. coli* is protected against oxidative stress by activating the SoxRS and the OxyR regulons [66, 67]. When *E. coli* K-12, grown in LB or defined media, is exposed to superoxide or redox-cycling drugs, the SoxR regulon activates the *soxS* gene that acts as transcription factor, inducing the expression of several genes from the SoxRS regulon. Some of the genes activated by the SoxS protein are *sodA*, *acnA*, *fumC*, *micF*, and *zwf*, replacing sensitive enzymes such as aconitase B and fumarases A and B with the oxygen resistant isozymes aconitase A and fumarase C [68, 69].

## Manipulation of sRNAs expression to improve high cell density *E. coli* growth in bioreactors

The identification of the different small RNAs associated with stress growth conditions, described in the previous section, was performed when the bacterial culture was grown on petri dishes or in shake flasks at low density. The research focused on discovering which sRNA responded to a specific stress condition and on locating potential target genes and their possible physiological roles. This was elucidated by using molecular biology and computational predictive tools. Since shake flasks are mostly being used for low density growth at non-controlled conditions, this approach did not provide sufficient information on the possible role of sRNAs on *E. coli* growth in bioreactor for biological production. At these growth conditions, especially when it is done to achieve high density growth, the bacteria are exposed to multiple stresses as a result of high nutrient and metabolite concentrations, changes in pH, dissolved oxygen, and temperature that can affect their growth and production capability [13]. Since some sRNAs were found to respond to bacterial stress, their overexpression or inhibition can potentially affect the bacterial growth and production properties especially at high cell density culture in a bioreactor. *E. coli* strains with modified sRNA expression will potentially be more resistant to stress and thus more suitable for industrial production. The following section summarizes the information currently available on utilizing sRNAs for creating *E. coli* strains less susceptible to environmental stresses, specifically pH, oxygen concentration, glucose, and acetate.

### Effect of SgrS manipulation on acetate in high cell density growth of *E. coli*

Growing *E. coli* to high cell densities is done, in most cases, by supplying the growing culture with adequate supply of glucose. However, glucose is also associated with acetate production that negatively affects the bacterial growth and, as a result, various growth strategies are routinely implemented, together with genetic construction of bacterial strains, to minimize acetate production and allow the bacteria to grow and produce without interruption [70, 71]. These growth strategies were focused on controlling the glucose supply rate, and on altering the central carbon metabolism by diverting the metabolic flux through increasing glucose uptake, manipulating the gene *cra* or modifying the phosphotransferase system (PTS). Although, these approaches solved the acetate accumulation they were associated with lower cell densities [72–76].

Several studies reported reduction in acetate excretion by knocking down the *ptsG* gene, which is responsible for coding the glucose-specific enzyme II of the PTS. This approach minimized the acetate excretion by reducing the glucose uptake which decreased the flux through glycolysis. However, the growth rate of this mutant strain was slower than the parental strain [72]. The genetic manipulation of specific genes by overexpression or deletion frequently limits cell growth since the regulation of other genes is being affected [3]. Using sRNAs for reducing acetate excretion is an attractive approach, since the regulation occurs at the posttranscriptional level and, therefore, might not affect cell growth.

**Fig. 1:**
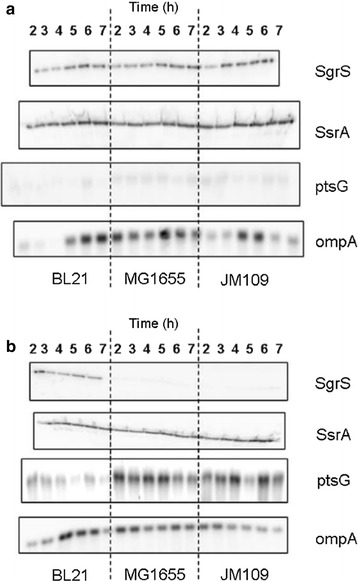
Transcription levels of sRNA and mRNA from *E. coli* growing in shake flasks. Transcription levels of the sRNAs SgrS, ssrA (internal control), mRNAs ptsG, and ompA (internal control) in *E. coli* B (BL21), *E. coli* K-12 (MG1655), and *E. coli* K-12 (JM109) grown in shake flask induced with **a** 10 g/L α-methyl glucoside and **b** 10 g/L glucose (Taken from [77])

When transcription of the sRNA SgrS was evaluated in *E. coli* culture growing in shake flask, its expression increased when the bacteria were exposed to the non-metabolizable glucose analog alpha methyl glucoside (αMG) which was associated with decreased expression of the glucose transporter ptsG, as seen in Fig. 1a [40, 77]. The increase in SgrS transcription and reduction in *ptsG* expression was observed in both *E. coli* B (BL21) and *E. coli* K-12 (JM109 and MG1655). However, when both strains were exposed to high glucose concentration, SgrS transcription was dissimilar; it increased in *E. coli B* and was not observed in *E. coli* K-12 (Fig. 1b). The increased SgrS transcription likely reduced the ptsG biosynthesis and lowered the bacterial glucose transport rate. This is an indication that *E. coli* B tolerates high glucose concentration, not only because of its more efficient central carbon metabolism [78] but also as a result of its ability to control the rate of glucose transport into the cells, thus minimizing the overflow of glucose and acetate production. The overexpression of SgrS in *E. coli* B was attributed to a possible accumulation of glucose-phosphate suggesting a possible overflow of glucose. This did not happen in *E. coli* K-12 perhaps because glucose was converted to acetate and pyruvate. Additionally, in high cell density cultures, the SgrS-*ptsG* interaction is activated by high glucose concentration and is specific to *E. coli* B.

**Fig. 2:**
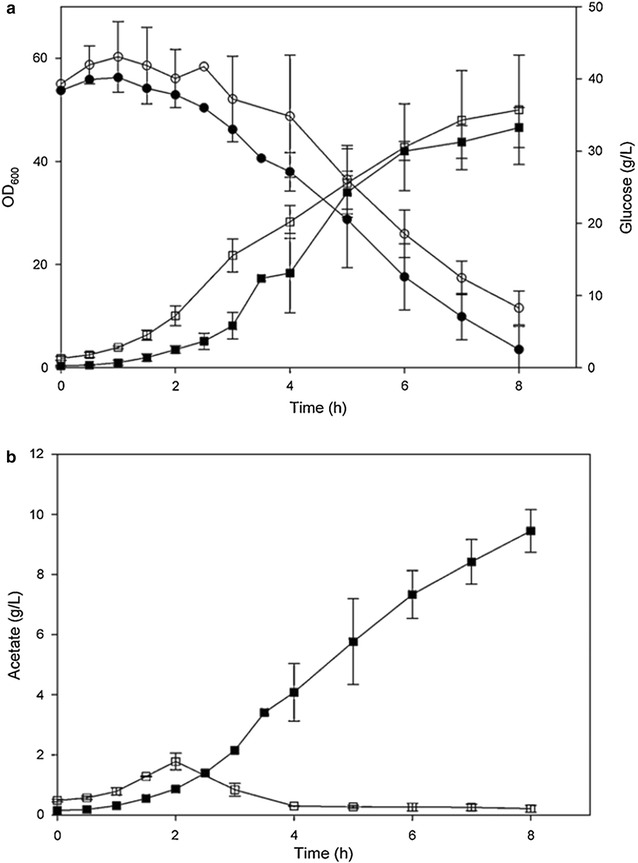
Growth, glucose consumption and acetate production in *E. coli* K-12(MG1655) and *E. coli* K-12 (MG1655 lacIq) containing plasmid pLCV1 overexpressing SgrS. **a** Growth and glucose consumption in (filled square, filled circle) *E. coli* K-12 (MG1655) and in (open square, open circle) *E. coli* K-2 (MG1655 lacIq) over-expressing SgrS. **b** Acetate excretion in (filled square) *E. coli* K-12 (MG1655) and in (open square) *E. coli* K-2 (MG1655 lacIq) over-expressing SgrS (Taken from [79])

Based on the above observations, an overexpression of the sRNA SgrS in *E. coli* K-12 seems to be a logical approach to increase the bacteria tolerance to high glucose concentration and to reduce acetate excretion. The behavior of *E. coli* K-12 overexpressing SgrS grown in bioreactor at high glucose concentration is seen in Fig. 2. Growth and glucose consumption in *E. coli* with plasmid control and with plasmid overexpressing SgrS described in Fig. 2a and acetate excretion of the two strains in Fig. 2b. It is obvious that SgrS expression minimized the negative effect of acetate accumulation on the cell growth, the low production of acetate without affecting cell growth suggest that the overexpressing SgrS strain is promising candidate for large scale production of recombinant proteins [79].

### Effect of GadY on growth of *E. coli* under acidic conditions

Exposure to low pH is a common stress condition during *E. coli* growth in media supplemented with glucose can affect the bacterial growth rate and protein production efficiency. The main cause for a drop-in pH is the accumulation of acetic acid [80], and it is usually occurring at high density growth. Even when the culture pH is being kept around 7.0 (by adding base, such as ammonium hydroxide or sodium hydroxide), there is always the possibility of local regions in the bioreactor where the pH is low, as was identified when dissolved oxygen concentration was followed in different sections of the bioreactor [81, 82]. Bacterial strains resistant to low pH will, therefore, have an advantage compared with the parental strain. Creating such strains by manipulating small RNA expression seems to be an attractive option, since, unlike genetic manipulation of glucose utilization pathways [75], it is possible that the effect on the bacterial growth and metabolism will be minimal.

**Fig. 3:**
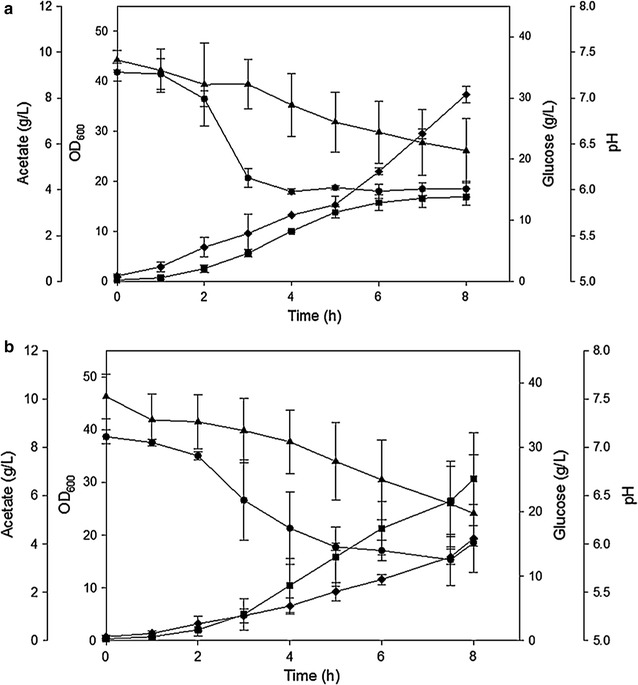
Cell growth parameters of *E. coli* MG1655 growing in bioreactor at pH 6.0 naturally decreased. **a** Parental strain and **b** GadY strain. (Square) OD_600_ (triangle) glucose (g/L) (diamond) acetate (g/L), and (circle) pH (Taken from [81])

As described in the previous section, the small RNA GadY was expressed at low pH in cultures grown in shake flasks [45] but no explanation for the GadY expression effect on the bacterial metabolism or growth was suggested. Expressing GadY was considered as a possible approach for creating a bacterial strain which may be less sensitive to low pH. To test this concept, an *E. coli* K-12 strain (MG1655) expressing the sRNA GadY was created and its performance in high cell density cultures in a bioreactor was compared with its parental strain [81]. It was found that at pH 7.0, both strains grew to a similar cell density of OD_600_ = 43, but the overexpressing GadY strain produced 40% less acetate than the parental strain. At pH 6.0, the parental strain (Fig. 3a) grew 35% less and produced 6 g/L (200%) more acetate than the GadY overexpressing strain (Fig. 3b) [81].

**Fig. 4:**
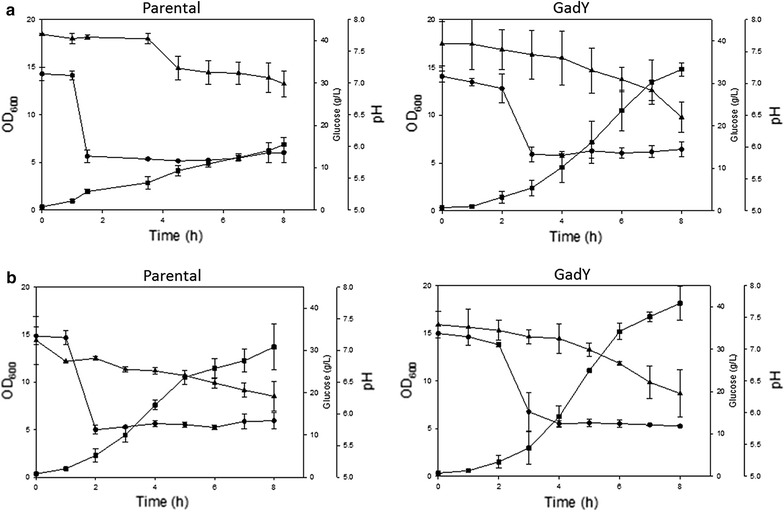
Cell growth parameters of *E. coli* K-12 MG1655 parental and GadY strains growing in bioreactor at pH 6.0. At OD 2.0 the pH was decreased from pH 7.0 to pH 6.0 by the addition of **a** acetic acid or **b** phosphoric acid. (Square) OD_600_ (triangle) glucose (g/L), and (circle) pH (Taken from [81])

The overexpressing GadY strain was evaluated further by following its response to media acidification with various organic acids [81]. The strain was exposed to pH 6.0 by adding acetic or phosphoric acid to the growth media (Fig. 4). The results showed that the overexpression GadY strain responded better than the parental strain to the acid addition and, that at the same pH, the acetic acid (Fig. 4a) affected the bacterial growth more than the phosphoric acid (Fig. 4b). Further analysis was performed by determining the expression level of 17 sRNAs that were previously reported to be associated with acid stress [83]. The results showed that in both strains growing at pH 7.0 the lysine decarboxylase system (LDS) was expressed in the early exponential phase and the glutamate decarboxylase system (GDS) was expressed in the late exponential phase. At pH 6.0, this pattern was similar except that the GDS was only expressed in the late exponential phase at the parental strain but not in the overexpressing GadY strain, an indication that GadY improved the growth of *E. coli* by deactivating the GDS system in the late exponential phase [81].

The overexpressing GadY strain was also found to decrease the production of acetate, regardless of the culture pH, which reduced further its negative effect on cell growth. Acetate dissociation depends on the media pH and affects the internal pH of the cells [80, 84]. Several processes and genetic approaches have been implemented to reduce acetate production, but some of these approaches had negative effects on cell growth and/or recombinant protein production [75]. An *E. coli* strain resistant to the acetate accumulation has an obvious advantage for high density growth and recombinant proteins production.

### Effect of OxyS on high cell density growth of *E. coli*

**Fig. 5:**
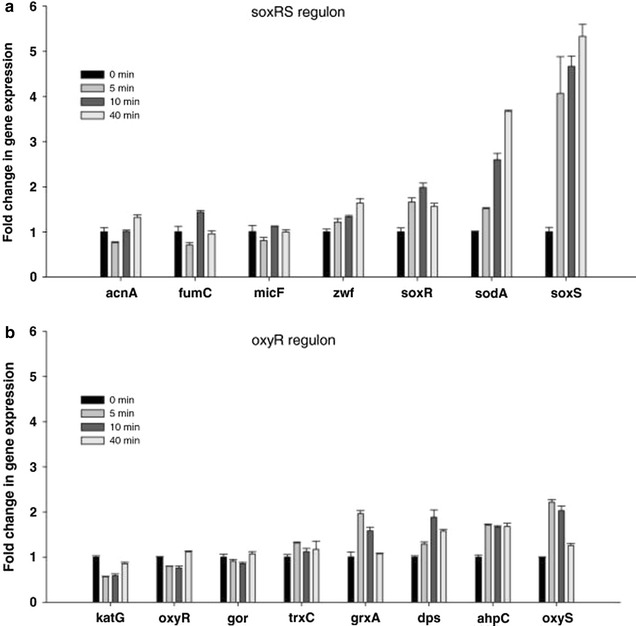
Time course expression of selected genes controlled by SoxRS and OxyR regulons during *E. coli* K-12 MG1655 growth at 30 and 300% dO_2_. **a** SoxRS controlled genes, **b** OxyR controlled genes, changes in mRNA were analyzed 0, 5, 10, and 40 min after dO_2_ shift from 30 to 300%. Error bars represent standard deviations between triplicate analyses (Taken from [90])

The small RNA OxyS is over-expressed when *E. coli* is exposed to oxidative stress generated by H_2_O_2_ or redox-cycling drugs, which the bacterial culture is not exposed to when it grown in shake flasks or in bioreactors [37, 69, 85–87]. To identify the possible role of this sRNA in high cell density cultures grown in bioreactor, its expression was followed when the bacterial culture was grown in the presence of high dissolved oxygen concentration. Supplying oxygen-enriched air or pure oxygen is a procedure commonly used to satisfy the oxygen demand of the bacterial culture [88, 89]. It was found that high concentrations of molecular O_2_ in a bioreactor caused the activation of the superoxide stress regulator SoxRS, which induces the transcription of the *soxS* and the *sodA* genes [90]. The *sodA* gene encodes the manganese superoxide dismutase (SOD), which allows the cells to continue growing during the oxidative stress [91]. The SoxRS and SOD system have been described as the major protection mechanisms against the toxic effects of pure O_2_. The main response of the cells to the oxygen stress was through the soxRS regulon (Fig. 5a) although there was a small increase in the OxyS expression, from the oxyR regulon (Fig. 5b) [90].

**Fig. 6:**
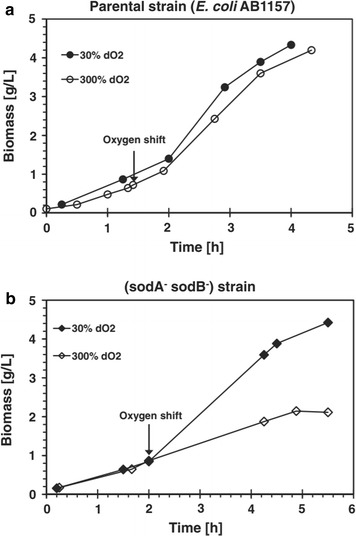
Effects of dissolved oxygen shift on growth of **a** *E. coli* AB1157 and **b** SOD deficient mutant. The arrows indicate when dO_2_ was increased from 30 to 300%. The 30% dO_2_ reference culture (circle) in (**a**) parental strain and (diamond) in (**b**) sodA^−^sodB^−^ strain (Taken from [90])

The OxyR, which activates the expression of the sRNA OxyS, is a transcriptional dual regulator responding to oxidative stress, mainly to high concentrations of H_2_O_2_. This regulon includes genes from the peroxide metabolism, redox balance, and manganese uptake [92, 93]. Since the OxyS was not activated while *E. coli* was grown at high dissolved oxygen concentrations (Fig. 6a), it is an indication that the H_2_O_2_ level was low, and the activation of the SoxRS system together with the SOD (Fig. 6b) was sufficient to protect the cells from the oxidative stress allowing the cells to continue growing without the contribution of OxyS [90]. The response to oxidative stress in high cell density cultures is an example where the posttranscriptional regulation mediated by this sRNA differs from the physiological response observed in low density cultures exposed to external induced stress.

## Summary

Changes in growth conditions are known to generate stress responses in *E. coli* by a variety of complex mechanisms, among them the posttranscriptional response mediated by small RNAs. By using experimental approaches, high-throughput computational searches [94], shotgun cloning [95] and tiling array analyses [22], 123 sRNAs have been experimentally verified and registered in the Rfam database and specifically for *E. coli* K-12 in the RegulonBD database [96, 97]. Although considerable work has been done on sRNA expression in *E. coli*, not much effort was directed towards utilizing small RNAs for creating *E. coli* strains resistant to growth-related stress conditions. Stress conditions are likely to occur when the bacteria are propagated at high density growth for production of biologicals. In this short review, we summarize the modifications of small RNA expression that were implemented to create *E. coli* strains resistant to stress conditions caused by glucose concentration, low pH, and pure O_2_. The strains overexpressing SgrS and GadY showed improved cell growth and an additional decrease in acetate productions. This indicates that manipulations at the posttranscriptional regulation, mediated by sRNAs, improve cell growth by enhancing cell resistance to low pH, and by decreasing the production of undesirable metabolites. This observation opens up ample opportunities to modify *E. coli* at the posttranscriptional level without affecting cell growth through the modifications of metabolic pathways. The current summary describes the effect of sRNAs only on glucose and pH, but there are certainly more stress-creating conditions that can affect the bacterial growth that need to be evaluated in industrial settings. We believe that the application of transcriptional regulation mediated by sRNA will improve large-scale productions of recombinant proteins in *E. coli* where environmental changes are likely to occur.

## Abbreviations

αMG: alpha methyl glucoside
FhlA: format hydrogen lyase activator
GDS: glutamate decarboxylase system
LDS: lysine decarboxylase system
SOD: manganese superoxide dismutase
MES: 2-(N-morpholino)ethanesulfonic acid
PTS: phosphotransferase system
ROS: reactive oxygen species
